# Characterization and Release Kinetics Study of Active Packaging Films Based on Modified Starch and Red Cabbage Anthocyanin Extract

**DOI:** 10.3390/polym14061214

**Published:** 2022-03-17

**Authors:** Meng Cheng, Xiaoran Yan, Yingjun Cui, Minjie Han, Yirong Wang, Juan Wang, Rongfei Zhang, Xiangyou Wang

**Affiliations:** School of Agricultural Engineering and Food Science, Shandong University of Technology, Zibo 255000, China; chengmeng0110@163.com (M.C.); xiaoranyan1222@163.com (X.Y.); cyj522819@163.com (Y.C.); hanminjie123@126.com (M.H.); wyrzhk8090@163.com (Y.W.); wangjuan7912@163.com (J.W.); zrf@sdut.edu.cn (R.Z.)

**Keywords:** active packaging, antioxidant activity, anthocyanin, release kinetics

## Abstract

Active packaging films were prepared by adding red cabbage anthocyanin extract (RCAE) into acetylated distarch phosphate (ADSP). This paper investigated the influence of the interaction relationship between RCAE and the film matrix on the structure, barrier, antioxidant and release properties of active films. Sixteen principal compounds in RCAE were identified as anthocyanins based on mass spectroscopic analysis. Micromorphological observations indicated that the RCAE distribution uniformity in the films decreased as the RCAE content increased. When the concentration of RCAE was not higher than 20%, the moisture absorption and oxygen permeability of films decreased. The stability of RCAE in the films was enhanced by the electrostatic interaction between RCAE and ADSP with the formation of hydrogen bonds, which facilitated the sustainability of the antioxidant properties of films. The release kinetics of RCAE proved that the release rate of RCAE in active films was the fastest in distilled water, and Fickian’s law was appropriate for portraying the release behavior. Moreover, the cytocompatibilty assay showed that the test films were biocompatible with a viability of >95% on HepG2 cells. Thus, this study has established the suitability of the films for applications in active and food packaging.

## 1. Introduction

In the food supply chain, packaging is an essential aspect to maintain food quality, reduce food waste and ensure food safety [1]. In traditional packaging, products are generally protected “passively” by using the barrier properties of the packaging materials [2,3]. Active packaging technology alters the function of the packaging from “passive” to “active”; that is, by adding antibacterial agents, antioxidants, releasing agents, absorbents and other active materials that control the internal environment of the package to better protect food [4,5]. Considering that oxidation is one of the major issues affecting food quality, the incorporation of antioxidants into packaging materials is promising, as they delay food spoilage [6,7]. With the increasing emphasis on food safety, adding natural antioxidants into packaging materials has become a research hotspot in recent years. Some of these natural antioxidants are plant essential oils [8], coconut shell extract [9], bamboo leaves [10], anthocyanin extract [11], and pine needles extract [12].

In recent years, the development of active packaging containing anthocyanins has become an important research area in the field of food engineering [13]. The results show that anthocyanins have strong antioxidant activity due to the presence of a large number of phenolic groups in their molecular structure [14,15]. Wang et al. [16] investigated the antioxidant activity of raspberry anthocyanin and reported that raspberry anthocyanin could effectively prevent the formation of 2,2′-Azinobis (3-ethyl-benzothiazoline-6-sulfonic acid) diammonium salt (ABTS) and 1,1-diphenyl-2-picrylhydrazyl (DPPH) free radicals. Due to the presence of the cyanidin 3,5-diglucoside, the majoritarian anthocyanin in red cabbage, this feedstock represents a promising source for obtaining anthocyanins in its concentrated form, which can be used as a natural antioxidant [17]. As the basic structure of red cabbage anthocyanin extract (RCAE) is cationic, they are highly susceptible to attack by reactive oxygen anions and free electrons, making them more unstable and susceptible to degradation [18]. In addition to their own structure, environmental factors, such as pH, temperature, light, metal ions, co-pigments and food additives, can also affect the stability of anthocyanins [19,20]. Therefore, it is of great importance to study and improve the stability of anthocyanins during processing and storage, so as to broaden their application scope.

Solid support is another component of active packaging, which plays an important role in fixing antioxidants, blocking the internal environment of packaging and protecting the packaged product. In recent years, some active films with antioxidant activity were prepared by adding anthocyanins or natural extracts into solid supports. Wang et al. [21] developed an antioxidant film by mixing gelatin with anthocyanins nanocomplexes for protecting olive oil from oxidation. The starch/PVA active film loaded with *Lycium ruthenicum* anthocyanins as packaging material can extend the shelf life of refrigerated fish [22]. Acetylated distarch phosphate (ADSP) is an esterified cross-linked composite-modified cassava starch obtained by acetylation and phosphate reactions of native cassava starch. Equally, ADSP films have good thermal stability and tensile strength. In our previous study, we illuminated that the stability of anthocyanins was enhanced by hydrogen bonding and electrostatic interactions between anthocyanin and ADSP [23]. Thus, this paper studied the effect of red cabbage anthocyanin extract (RCAE) as an antioxidant on the properties of active packaging films. By regulating the concentration of the active ingredient (RCAE) in order to adjust such important parameters as moisture absorption and oxygen permeability, which are crucial in the packaging of food products. The work conducted research on physicochemical properties, including morphology, optical and thermal properties, oxygen permeability, moisture absorption, antioxidant properties of the active films. Moreover, the release behavior of RCAE in ADSP-based active films was simulated in four different packaging environments. To ensure consumers safety, the biocompatibility of the active films containing RCAE was assessed on a human liver cancer cell line (HepG2).

## 2. Materials and Methods

### 2.1. Materials

Acetylated distarch phosphate (ADSP) was purchased from Dongguan Dongmei Food Co., Ltd. (Guangdong, China). Anthocyanins were extracted from red cabbage (Shandong, China) based on our previous study [23]. Glycerol, absolute ethanol, thiazolyl blue tetrazolium bromide (MTT) and dimethyl sulfoxide (DMSO) were from Sinopharm Chemical Reagent Co., Ltd. (Shanghai, China), and all were of analytical reagent grade. Dulbecco’s modified Eagle’s medium (DMEM) was purchased from Gibco (Grand Island, NY, USA). The human liver cancer (HepG2) cell line was obtained from the cell bank of the Institute of Biochemistry and Cell Biology, Chinese Academy of Sciences (Shanghai, China).

### 2.2. Characterization of Red Cabbage Anthocyanin Extract (RCAE)

The components of RCAE were analyzed by the Agilent 1200 HPLC system (Agilent Technologies, Santa Clara, CA, USA) equipped with a 6460 triple quadrupole mass spectrometer according to the methods of Yong et al. [24] and Liu et al. [25]. The data were acquired by the Q-Exactive system using Xcalibur 4.1 software (Thermo Fisher Scientific, Waltham, MA, USA) and processed by TraceFinder™4.1 clinical software (Thermo Fisher Scientific, Waltham, MA, USA). The quantified data were output into the Excel format.

### 2.3. Preparation of Active Films

In brief, 4 g of ADSP was dissolved and gelatinized in 100 mL of distilled water containing 1.2 g of glycerol as a plasticizer at 90 °C. When the mixture was cooled to 25 °C, RCAE (131.2 g/L) was added to the above solution to obtain final concentrations of 0, 10%, 20%, 30% and 40% (*w*/*w*), which were designated as S0, S1, S2, S3 and S4, respectively. Then, the film-forming solutions were poured into a plastic mold of equal dimensions (12 cm × 12 cm) to prepare the films and dried in an oven at 45 °C. The films were conditioned (temperature, 25 °C; relative humidity (RH), 50%) for 2 days before commencement of the test.

### 2.4. Film Characterization

#### 2.4.1. Microstructural Characteristics

The surface morphology of the film samples was revealed by scanning electron microscopy (SEM; JEOL, Tokyo, Japan). The samples were sputtered with gold before the analysis [26]. The surface of the film (size, 15 μm × 15 μm) was photographed by atomic force microscopy (AFM; Nanoscope V, Bruker, MA, USA) and the 3D images of the film were constructed. The spatial resolution of the microscope was 1 nm in the transverse direction and 0.1 nm in the longitudinal direction, and the scanning speed was 1 Hz. The average roughness (Ra) and root mean square roughness (Rq) of the surface of films was calculated by NanoScope Analysis 1.7 software as previously described [27,28].

#### 2.4.2. Thermogravimetric Analysis

The thermal stability of the films was analyzed by heating films at a rate of 10 °C/min (inert atmosphere: 50 mL/min of N_2_). The thermal analyses were performed at temperatures ranging from 26 °C to 600 °C [29].

#### 2.4.3. Color Properties and Opacity

The color of the films was evaluated using a portable colorimeter (CR410, Konica Minolta Sensing, Inc., Sakai Osaka, Japan). A white standard color plate was used as a standard during the color measurement, and lightness (L*) and chromaticity parameters a* (red–green) and b* (yellow–blue) were used to characterize the film color. Total color difference (Δ*E*) was calculated using the following equation [6]:

$$\Delta E=\sqrt{{\left(L-L^{*}\right)}^{2}+{\left(a-a^{*}\right)}^{2}+{\left(b-b^{*}\right)}^{2}}$$

where *L*, *a*, and *b* are the color values of the standard color plate.

The film sample (size, 8 mm × 40 mm) was recorded at 600 nm by the Lambda 35 UV–vis spectrophotometer (PerkinElmer Inc., Waltham, MA, USA). The opacity of each film was determined by the method of Hasheminya et al. [30].

$$Opacity=\frac{A_{600}}{X}$$

where *X* is the film thickness (mm), and *A*_600_ is the absorbance of film at 600 nm.

#### 2.4.4. Oxygen Permeability (OP)

The oxygen permeability (OP) of films was measured by an oxygen permeability test system (CLASSIC 216, Labthink Technology Co., Ltd., Jinan, China). All samples were determined at a temperature of 25 °C and a RH of 75% as previously described [31].

#### 2.4.5. Moisture Absorption

The moisture absorption of the films was determined according to the method of Prachayawarakorn and Kansanthia [32]. The films (size, 25 mm × 25 mm) were desiccated at 105 °C for 6 h and weighed (*W*_1_). Next, the films were incubated at a RH of 99 ± 1% for 10 days (*W*_2_). The moisture absorption percentage was calculated as follows:

$$\mathrm{Moisture}\;\mathrm{absorption}\;\left(\%\right)=\frac{W_{2}-W_{1}}{W_{1}}\times 100\%$$

#### 2.4.6. Antioxidant Activity

The antioxidant activity of films was evaluated by the 1,1-diphenyl-2-picrylhydrazyl (DPPH) free radical scavenging assay [33] using a reagent test kit (Solarbio Science & Technology Co., Ltd., Beijing, China). In brief, 0.05 g of the film sample was weighed and added to 1 mL of extraction solution for determination. The antioxidant activity of films stored at 25 °C for 0 and 7 days was determined.

#### 2.4.7. Release Behavior

A release test of RCAE from the films was performed as previously described [34,35,36]. S4 samples (size, 6 cm × 6 cm) were placed in the brown bottles containing 20 mL of distilled water, 10%, 50% and 95% ethanol solutions. The samples were gently shaken at 100 rpm (25 °C) under dark conditions. The absorbance of sample solutions was determined by a UV spectrophotometer. The in vitro release data were fitted to the release kinetic model to understand the effects of the relationship between RCAE and the ADSP matrix.

#### 2.4.8. Cytotoxicity Assay

The cytotoxicity of ADSP-based films on HepG2 cells was evaluated by the MTT assay [37]. HepG2 cells were inoculated at a density of 1 × 10^4^ cells/mL and cultured in 96-well plates containing DMEM for 24 h. The fresh medium was readded after the old medium had been sucked out. For the experimental group, the film samples (S0–S4; diameter, 8 mm) were placed at the bottom of the hole to directly contact HepG2 cells, while no films were used for the blank control group. After 48 h of treatment, the film samples were removed and washed with PBS for three times. Next, 100 μL of MTT (final concentration, 0.5 mg/mL) was added to each well, and the plates were incubated for 4 h. The medium containing MTT was aspirated, and 200 μL of DMSO was added to each well to dissolve the formamide crystals. The absorbance at 570 nm was measured, and the blank control group was used to indicate 100% cell viability group.

### 2.5. Statistical Analysis

All analyses were run in multiples and the results were expressed as means ± standard deviation. Analysis of variance (ANOVA) and Duncan’s new multiple range test were performed at a significance level of *p* < 0.05 using SPSS 19.0 (IBM, Armonk, NY, USA).

## 3. Results and Discussion

### 3.1. Identification and Relative Quantification of RCAE

Sixteen anthocyanin structures were identified in RCAE (Table 1). Notably, cyanidin 3,5-diglucoside was characterized as the predominant component, which accounted for 53.913% of the total anthocyanins, followed by delphinidin (31.786%), cyanidin 3-galactoside (5.210%), cyanidin-3-(sinapoyl) (sinapoyl)-diglycoside-5-glycoside (4.611%) and petunidin (2.840%). The other compounds identified in RCAE constituted less than 2% of the total anthocyanins. The components of anthocyanins characterized in this study were somewhat different from those of previous reports [38,39], which could be due to the different varieties, genotypes, cultivation regions and growth conditions of red cabbage. The relative quantification by cyanidin 3,5-diglucoside and the relative content observed for each compound in the LC-MS analysis are illustrated in Table 1. Notably, RCAE contained acylated molecules (cyanidin-3-(sinapoyl) (sinapoyl)-diglycoside-5-glycoside).

### 3.2. Morphology and Structure Analysis

**Figure 1:**
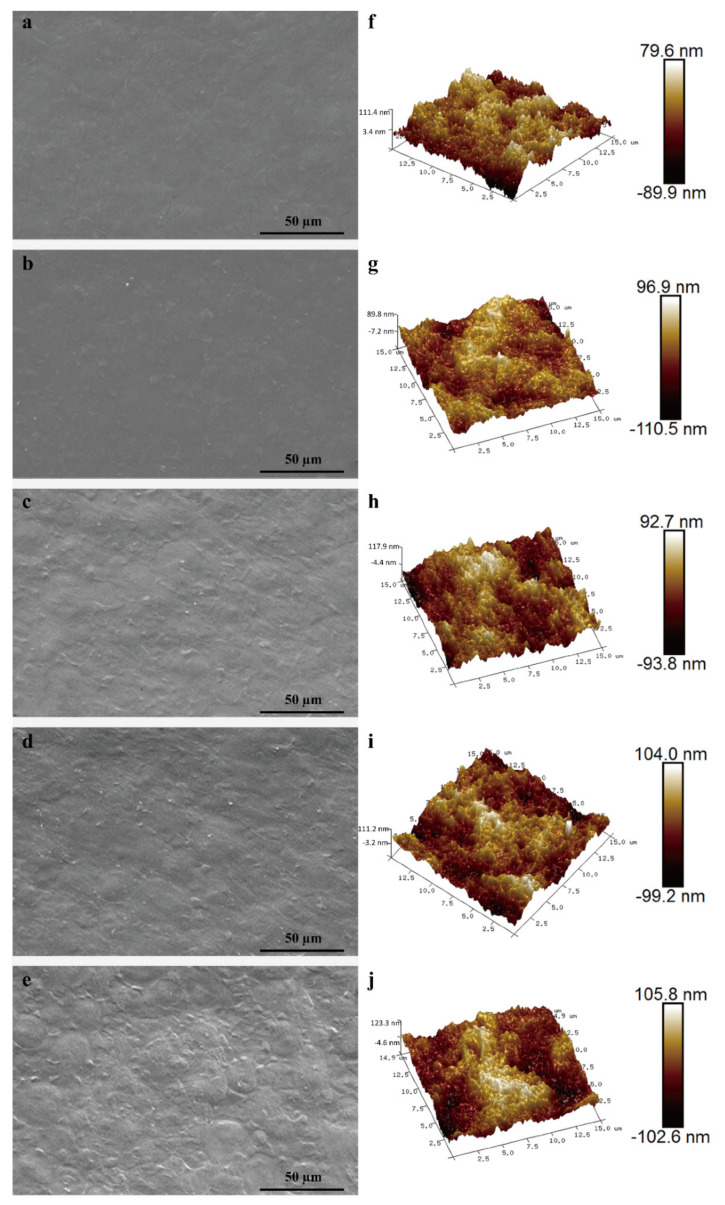
SEM (**a**–**e**) and AFM (**f**–**j**) images of active films with different RCAE contents: S0 (**a**,**f**), S1 (**b**,**g**), S2 (**c**,**h**), S3(**d**,**i**), and S4 (**e**,**j**).

The microstructure of films was investigated by SEM to further explain the relationship between structures of the films and related properties. The surface of S0 films was uniform and smooth (Figure 1a). By contrast, the films incorporated with RCAE showed rougher surfaces (Figure 1b–e), which was indicative of the uneven mixing of ADSP, RCAE and glycerol in the film. The poor distribution of the anthocyanins in the films produced by casting could be related to the convective flow during drying that lifts the particles to the surface and to the poor chemical affinity between anthocyanins and the polymer matrix. Similar observations have been confirmed earlier [40]. Vedove, Maniglia and Tadini [41] also found that the surface of the cassava starch-based films was also roughened by the addition of anthocyanins.

The 3D topography of active films was performed by AFM, as shown in Figure 1f–j. The AFM images of S0 films showed revealed a smooth surface with lower Ra and Rq values of 17.7 nm and 22.4 nm, respectively (Table 2). The images of S2 films exhibited a hill-valley structure with Ra and Rq values of 21.0 nm and 26.3 nm, respectively. Similarly, the images of S4 films also depicted an irregular surface with Ra and Rq values of 26.3 nm and 32.3 nm, respectively (Table 2). Adding immiscible filler particles gave higher variation in surface height and roughness of the films. The roughness differences between S0 and S4 films were consistent with the SEM observations. In fact, too much RCAE was incorporated into the ADSP polymer, which was not conducive to the dispersion of the films, resulting in the formation of aggregates in the films. Zhang et al. [42] testified that aggregates appeared on the surface of sodium carboxymethyl starch/κ-carrageenan films after the addition of the anthocyanin extract. In addition, the surface roughness of gelatin films increased with the incorporation of anthocyanins in the films [21]. Eze et al. [43] also reported similar findings.

### 3.3. Thermal Analysis

**Figure 2:**
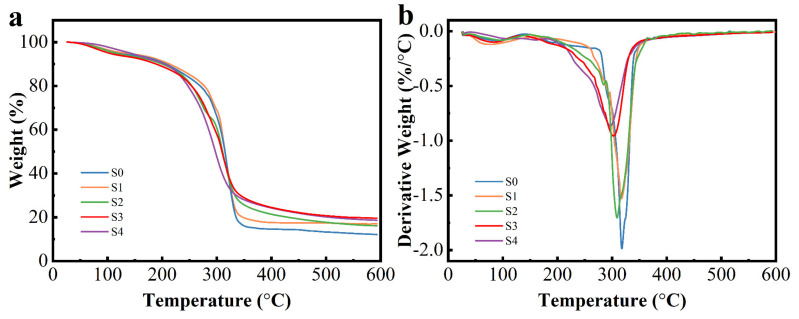
TGA (**a**) and DTG (**b**) curves of ADSP-based films added by different RCAE contents.

The thermal stability of the films was checked by the TGA and DTG curves. The TGA and DTG thermograms of S0, S1, S2, S3 and S4 films are shown in Figure 2. The first stage (30–105 °C) was attributed to the evaporation of loosely bound water in films [44]. The next reduction in mass, which was observed around 105–249 °C, corresponded to the decomposition of glycerol [45]. Finally, the final weight loss between 250–460 °C was related to the polymer degradation in the films [46]. As shown in Figure 2b, the peak of maximum degradation decreased as the RCAE amount increased. This behavior was probably due to the decomposition of volatile molecules present in the RCAE [27]. Prietto et al. [39] found similar results for starch films incorporated with anthocyanins, suggesting less thermal stability of the films. The findings were also reported by Freitas et al. [27], whose cellulose acetate films displayed less thermal stability in the film when incorporated with red cabbage extract. In addition, this result could be related to the interaction weakening in the polymer chains when RCAE was added, which facilitated its decomposition at lower temperatures. As shown in Figure 2b, the S0 films exhibited a peak maximum at 318 °C, whereas the ADSP-RCAE films presented peak maximum at 297–316 °C. The corresponding maximum temperature for the thermal degradation of S4 films was only 21 °C lower than that of S0 films. These results indicated that the active films remain stable at 200 °C, which was suitable for most of the food packaging applications.

### 3.4. Color Properties and Opacity

The packaging films should possess the barrier ability of light to prevent the oxidation of food. The optical properties of films depend on their microstructure, which are affected by the surface and internal heterogeneity of the structure [47]. As demonstrated in Table 3, after incorporating the RCAE in the active films the opacity improved with an enhancement in the RCAE content (*p* < 0.05), indicating that RCAE could increase the opacity of ADSP-based films. These results were in accordance with the color of the active films. After the addition of the RCAE, the values of L* and b* reduced, and a* and ΔE significantly increased (*p* < 0.05). This indicated that the films became redder and had higher opacity. This phenomenon may be caused by the original color of the RCAE. Notably, the covered pattern can be clearly observed in each composite film (Figure S1), indicating that the increase in the opacity of the composite film did not affect the observation of packaged food. In addition, morphological and structural analyses revealed that the surface of the films incorporated with RCAE was rougher, which resulted in the formation of the non-uniform film surface. Incorporated RCAE partially replaced void space in the film matrices where light passed through, giving lower light transmission [48,49]. Additionally, the opacity of the S4 film reached 1.342. Wang et al. [50] also reported that the incorporation of black soybean seed coat extract could greatly reduce the transparency of chitosan films. Bai et al. [51] also proved similar findings.

### 3.5. Oxygen Permeability

As shown in Table 2, the OP value of films decreased along with an increase in RCAE content from 0% to 20% (*w*/*w*). The –OH from RCAE and ADSP promoted the formation of intermolecular hydrogen bonds, resulting in highly dense and cohesive films. These findings were consistent with a previous study [23]. Wu et al. [49] demonstrated that the incorporation of black rice bran anthocyanins extract decreased the oxygen barrier properties of the active films. However, the OP value of the S4 films significantly increased to 4.31 × 10^−14^ cm^2^ s^−1^ Pa^−1^ (*p* < 0.05). With the gradual increase in RCAE content, the free positive charges prevented the electrostatic attraction between ADSP and anthocyanin. The dense network structure of films was loosened, thus expanding the free volume inside the molecular and facilitating the movement of the molecular chains [27,52]. Moreover, the surface of the films became rougher after the addition of RCAE, which resulted in the non-uniform film surface leading to faster diffusion of gas molecules, which ultimately led to an increased in the oxygen permeability (OP) of the ADSP-based films. Freitas et al. [27] also discovered that the oxygen permeability of cellulose acetate-based films could increase.

### 3.6. Moisture Absorption

Moisture adsorption mainly reflects the uptake by the film matrix, which is usually used to assess the water resistance of a film [53]. Generally, the lower value of these indexes indicate the better water resistance of the film [54]. The moisture absorption test was performed at a RH of 99 ± 1% for 10 days, and the data are shown in Table 2. The moisture absorption of S0 films was 20.36%, but as the RCAE increased from 10% to 20%, the moisture absorption of the films significantly decreased from 18.14% to 16.98% (*p* < 0.05), which could be related to the electrostatic interactions and hydrogen bonding between RCAE and ADSP. Chi et al. [55] also reported similar findings, which testified that the moisture absorption of ĸ-carrageenan/hydroxypropyl methylcellulose composite films showed a decreasing trend after the addition of grape skin powder. However, the moisture absorption of S4 films increased to 23.07%, and the increasing proportion of –OH groups at higher concentrations of RCAE was the reason for the increased moisture absorption of S4 films. The absorption of moisture by the film surface was conducive to the release of anthocyanins from ADSP and the formation of –OH groups, which controlled the release rate of RCAE in active films.

### 3.7. Antioxidant Activity

**Figure 3:**
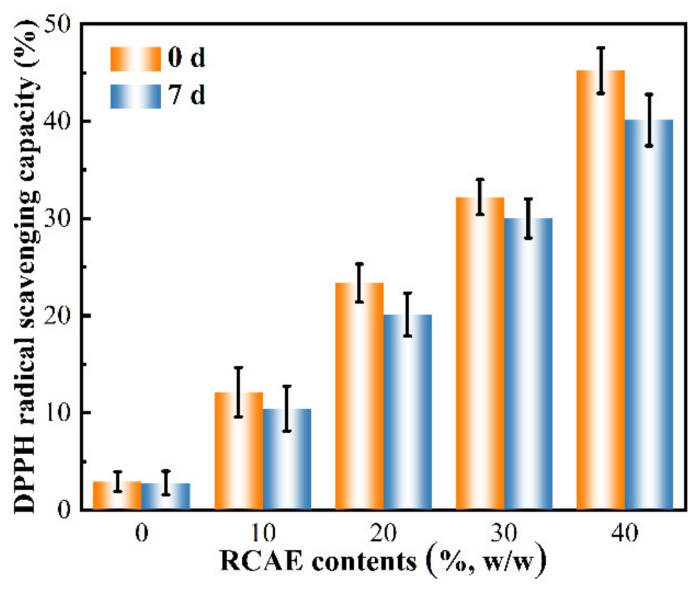
The DPPH radical scavenging capacity of ADSP-based films added by different RCAE contents stored 0 and 7 days placed at 25 °C.

The antioxidant activity of active films was determined by the DPPH radical scavenging capacity. The antioxidant activity could change the color of the DPPH radical into the yellow diphenylpicrylhydrazine complex. The degree of this reaction mostly depends on the hydrogen donating ability of the antioxidant. As shown in Figure 3, the S0 films showed approximately 2.92% of antioxidant activity. The addition of RCAE could significantly improve the scavenging activity against DPPH of active films, which was mainly ascribed to phenolic compounds in RCAE that showed strong antioxidant capacity [43]. The DPPH radical scavenging capacity of the S4 films (time 0 days) reached 45.21 ± 2.33%. These findings indicate that the addition of RCAE enhanced the antioxidant activity of ADSP films, which was mainly due to the strong hydrogen donating ability of RCAE that reduced the activity of the radicals or completely eliminated them [9,56]. These findings reveal the potential application of ADSP films containing RCAE in antioxidant packaging, as well as the very promising development in functional packaging. Mushtaq et al. [57] found that films with the addition of pomegranate peel extract retarded the oxidation of fats and proteins in cheese. Other studies demonstrated that an increase in phenolic hydroxyl content is critical for improving antioxidant activity [58]. When the active films were stored for 7 days, the DPPH radical scavenging capacity of films decreased slightly compared with that of films stored for 0 days (*p* > 0.05), indicating that RCAE has good stability in ADSP-based films. Interestingly, this was possibly because of the presence of acylated anthocyanin, which can confer better stability to the molecules [59]. Moreover, this may be the result of the formation of stable complexes of RCAE with ADSP through hydrogen bonding and electrostatic interactions, which improved the stability of RCAE.

### 3.8. Release Kinetics

**Figure 4:**
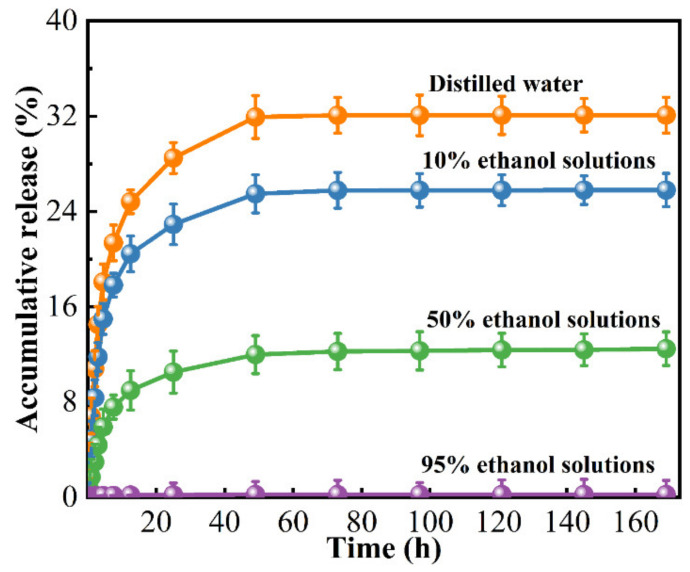
Accumulative release of RCAE from S4 films.

The profiles of kinetic release of RCAE from the ADSP-based active films into simulants in four different packaging environments are presented in Figure 4. The S4 films presented the same behaviour in all simulated solutions, with a burst release in the first 25 h and stabilization afterwards. It is worth noting that the release of RCAE from the films was 0 at the initial moment. When the release time was 169 h, the accumulative release of RCAE from ADSP-based films in distilled water, 10%, 50% and 95% ethanol solutions was 32.08%, 25.79%, 12.48% and 0.23%, respectively. The release of RCAE from the S4 films after 73 h of immersion in distilled water reached equilibrium, showing more than twice as much as in the 50% alcohol solution. These findings revealed that the release rate of RCAE in films was the fastest in distilled water, followed by 10% and 50% alcohol solutions, and RCAE was released slowest in the 95% alcohol solution. In general, the release of active compounds is influenced by the water solubility of the polymer, swelling rate, type of extracted food simulant and diffusion of active compounds from the film to the simulant [60]. The high swelling of films in water and the high solubility of RCAE in water could have promoted in the fast release of RCAE in distilled water. On the contrary, the low solubility of RCAE in alcohol solutions and the insolubility of the ADSP-based films in ethanol solutions could have facilitated the slower release of RCAE from the films in other simulants. Ezati and Rhim [61] reported that the release rate of the films was higher in 50% ethanol solutions than that in water, as well as in 10% and 95% ethanol solutions, and that the release rate of alizarin was solution dependent.

The cumulative release curves can provide valuable information on the release mechanisms and kinetics. The application of the Korsmeyer–Peppas model for the fitness of experimental data pertaining to the release of RCAE from ADSP-based films is shown in Table 4, indicating good predictive power (correlation coefficients (R^2^) > 95%). Similarly, Singh et al. [62] demonstrated that the release kinetics of anthocyanin in chitosan/poly(vinyl alcohol) films was best fitted to first-order kinetics from the coefficient of the correlation-fitted data. The results of our investigation showed that Fickian diffusion was suitable for the simulation of RCAE release from ADSP-based films in the four different packaging environments, as evidenced by n values less than 0.5 [63]. Wu et al. [64] reached a similar conclusion. The diffusion property of RCAE from films would be conducive to their sustainable antioxidant activity.

### 3.9. Cytotoxicity Assay

The materials used for food packaging are supposed possess non-toxic characteristics. In the experiment, we found that the cell viability was approximately 95% after 48 h of direct contact between all films and cells (*p* > 0.05). In vitro cytotoxicity studies revealed that these active films were non-cytotoxic packaging materials, in accordance with ISO 10993-5:2009 [65]. Similarly, Andonegi et al. [66] confirmed that chitosan/collagen films were non-cytotoxic biomaterials.

## 4. Conclusions

In summary, the active films were prepared by the addition of RCAE to ADSP as the film substrate. Morphological and structural analyses showed that the surface of the films became rougher after the addition of RCAE, which caused an uneven film surface. Additionally, the highest average roughness of the S4 films was 26.3 nm. The addition of RCAE improved the light, oxygen barrier properties and water-resistance of films. In addition, these films showed enhanced thermal stabilities and antioxidant activities. These enhancements were attributed to the electrostatic interaction between RCAE and ADSP with the formation of hydrogen bonds. The release kinetics of RCAE proved that the release rate of RCAE in the films was the fastest in distilled water, and Fickian’s law was appropriate for portraying the release behavior. Moreover, these active packaging films also showed great biocompatible capabilities. As such, our findings indicated that the addition of RCAE could improve the physical performance of the ADSP-based films and the composite films had good application potential as packaging films.

## Figures and Tables

**Table 1:** Characteristics of anthocyanins found in RCAE with their relative amounts.

Compound	Molecular Formula	[M+H]+ (*m*/*z*)	Retention Time (min)	Relative Quantification (ng/mL)	Relative Content (%)
Cyanidin 3,5- diglucoside	C_27_H_31_O_16_	611.16	2.66	6938.769 ± 23.841	53.913 ± 0.185
Cyanidin 3- galactoside	C_21_H_21_O_11_	449.11	3.89	670.568 ± 10.772	5.210 ± 0.084
Cyanidin-3-(sinapoyl)(sinapoyl)-diglicoside-5-glicoside	C_43_H_64_N_2_O_36_	1185	4.24	593.481 ± 11.219	4.611 ± 0.087
Delphinidin	C_15_H_11_O_7_	303.05	4.71	4090.924 ± 16.021	31.786 ± 0.124
Procyanidin B4	C_30_H_26_O_12_	579.15	5.35	7.710 ± 0.318	0.060 ± 0.002
Cyanidin	C_15_H_11_O_6_	287.06	5.39	7.444 ± 0.521	0.058 ± 0.004
Procyanidin B2	C_30_H_26_O_12_	579.15	5.46	0.720 ± 0.053	0.006 ± 0.004
Petunidin	C_16_H_13_O_7_	317.07	5.57	365.524 ± 5.647	2.840 ± 0.044
Pelargonidin	C_15_H_11_O_5_	271.06	6.02	0.866 ± 0.524	0.007 ± 0.004
Malvidin	C_17_H_15_O_7_	331.08	6.21	4.278 ± 0.306	0.033 ± 0.002
Peonidin	C_22_H_23_O_11_	301.07	6.18	67.232 ± 3.499	0.522 ± 0.027
Rutin	C_27_H_30_O_16_	611.16	6.78	3.610 ± 0.041	0.028 ± 0.001
Luteolin	C_15_H_10_O_6_	287.06	8.17	37.580 ± 2.314	0.292 ± 0.018
Quercetin	C_15_H_10_O_7_	303.05	8.19	50.118 ± 2.597	0.389 ± 0.020
Isorhamnetin	C_16_H_12_O_7_	317.07	8.36	14.548 ± 1.573	0.113 ± 0.012
Kaempferol	C_15_H_10_O_6_	287.06	8.35	16.808 ± 3.112	0.131 ± 0.024

**Table 2:** Roughness parameters, barrier properties, moisture absorption and cell viability of active films with different RCAE contents.

Film Type	Ra (nm)	Rq (nm)	OP (×10^−14^ cm^2^ s^−1^ Pa^−1^)	Moisture Absorption (%)	Cell Viability (%)
S0	17.7 ± 0.2 ^a^	22.4 ± 0.4 ^a^	2.43 ± 0.13 ^a^	20.36 ± 0.29 ^a^	97.04 ± 0.31 ^a^
S1	18.8 ± 0.6 ^b^	23.5 ± 0.3 ^b^	2.06 ± 0.18 ^b^	18.14 ± 0.23 ^b^	96.38 ± 0.52 ^ab^
S2	21.0 ± 0.8 ^c^	26.3 ± 0.5 ^c^	1.71 ± 0.07 ^c^	16.98 ± 0.31 ^c^	95.69 ± 0.78 ^b^
S3	24.0 ± 0.4 ^d^	30.2 ± 0.2 ^d^	1.78 ± 0.09 ^c^	17.05 ± 0.26 ^c^	95.72 ± 0.15 ^b^
S4	26.3 ± 0.7 ^e^	32.3 ± 0.6 ^e^	4.31 ± 0.20 ^d^	23.07 ± 0.34 ^d^	95.59 ± 0.27 ^b^

The values are presented as means ± SD. Different letters within the same column indicate significant differences (*p* < 0.05).

**Table 3:** Color and opacity of active films with different RCAE contents.

Film Type	L*	a*	b*	Δ*E*	Opacity (mm^−1^)
S0	96.15 ± 0.06 ^a^	−1.10 ± 0.02 ^a^	1.88 ± 0.11 ^a^	0.76 ± 0.09 ^a^	0.395 ± 0.010 ^a^
S1	93.67 ± 0.18 ^b^	1.91 ± 0.23 ^b^	−0.09 ± 0.01 ^b^	4.08 ± 0.17 ^b^	0.580 ± 0.010 ^b^
S2	89.50 ± 0.43 ^c^	4.92 ± 0.61 ^c^	−1.72 ± 0.52 ^c^	9.25 ± 0.77 ^c^	0.752 ± 0.004 ^c^
S3	86.52 ± 0.39 ^d^	7.36 ± 0.18 ^d^	−3.42 ± 0.14 ^d^	14.53 ± 1.22 ^d^	0.781 ± 0.007 ^d^
S4	82.74 ± 0.06 ^e^	11.20 ± 0.20 ^e^	−6.68 ± 0.78 ^e^	23.07 ± 3.00 ^e^	1.342 ± 0.009 ^e^

The values are presented as means ± SD. Different letters within the same column indicate significant differences (*p* < 0.05).

**Table 4:** Parameters of Korsmeyer–Peppas model at 25 °C for release.

Simulated Solutions	K	n	Correlation Coefficients (R^2^)
Distilled water	0.356 ± 0.009	0.332 ± 0.007	0.969 ± 0.001
10% ethanol aqueous	0.303 ± 0.003	0.333 ± 0.010	0.958 ± 0.001
50% ethanol aqueous	0.242 ± 0.002	0.388 ± 0.006	0.963 ± 0.001
95% ethanol aqueous	0.196 ± 0.003	0.426 ± 0.009	0.984 ± 0.003

The values are presented as means ± SD.

## Data Availability

The data presented in this study are available on request from the corresponding author.

## Supplementary Materials

The following supporting information can be downloaded at: https://www.mdpi.com/article/10.3390/polym14061214/s1, Figure S1: Physical appearances of intelligent packaging films on the white papers.
