# Supplementary material for: Characterization and Release Kinetics Study of Active Packaging Films Based on Modified Starch and Red Cabbage Anthocyanin Extract
Source: Polymers (Basel). 2022 Mar 17;14(6):1214. doi: 10.3390/polym14061214 (PMC8950823; doi:10.3390/polym14061214)
Supplement: Supplementary file 1 [file polymers-14-01214-s001.zip › polymers-1627211-supplementary.pdf]

Supplementary Material

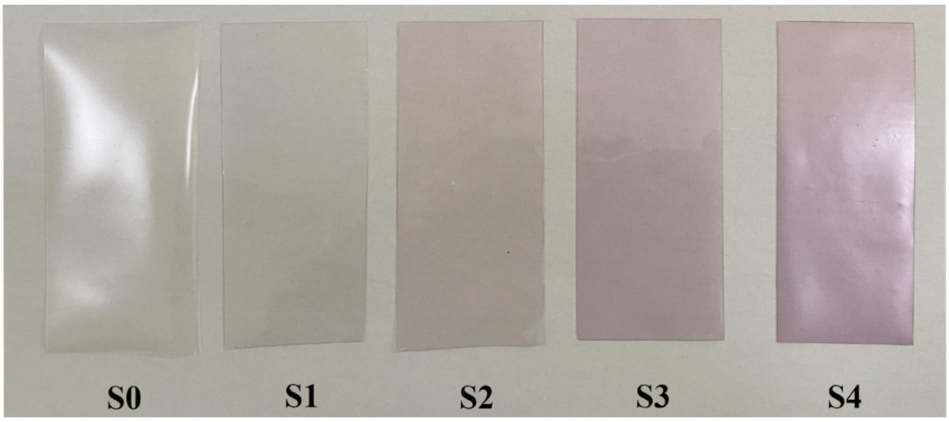

Figure S1. Physical appearances of intelligent packaging films on the white papers.
